# Using a Standing Heel-Rise Test as a Predictor of Ankle Muscle Strength in the Elderly

**DOI:** 10.3390/sports11080146

**Published:** 2023-08-02

**Authors:** Weerasak Tapanya, Sinthuporn Maharan, Noppharath Sangkarit, Puttipong Poncumhak, Saisunee Konsanit

**Affiliations:** 1 Department of Physical Therapy, School of Allied Health Sciences, University of Phayao, Phayao 56000, Thailand; sinthuporn.ma@up.ac.th (S.M.); noppharath.sa@up.ac.th (N.S.); puttipong.po@up.ac.th (P.P.); saisunee.ko@up.ac.th (S.K.); 2 Unit of Excellence of Human Potential Development and Herbs, University of Phayao, Phayao 56000, Thailand

**Keywords:** isometric dynamometer, older adults, demographic data, balance, prediction

## Abstract

The senior population is at increased risk of falling due to a reduction in ankle muscle strength. Evaluating the strength of the ankle muscles in older adults is of paramount importance. The purpose of this study was to formulate an equation to estimate ankle muscle strength by utilizing the basic physical characteristics of the subject and the variables related to their ability to perform the standing heel-rise test (SHRT). One hundred and thirty-two healthy elderly participants (mean age 67.30 ± 7.60) completed the SHRT and provided demographic information. Ankle plantar flexor (PF) muscle strength was evaluated using a push–pull dynamometer. Multiple regression analysis was utilized to develop a prediction equation for ankle PF muscle strength. The study revealed that the ankle PF strength equation was derived from variables including the power index of the SHRT, gender, age, calf circumference, and single-leg standing balance test. The equation exhibited a strong correlation (r = 0.816) and had a predictive power of 65.3%. The equation is represented as follows: ankle PF strength = 24.31 − 0.20(A) + 8.14(G) + 0.49(CC) + 0.07(SSEO) + 0.20(BW/t-SHRT). The equation had an estimation error of 5.51 kg. The strength of ankle PFs in elderly individuals can be estimated by considering demographic variables, including gender, age, calf circumference, single-leg standing balance test, and the power index of the SHRT. These factors were identified as significant determinants of ankle PF strength in this population.

## 1. Introduction

Muscle strength and power are essential for maintaining balance in the elderly. Balance is the ability to maintain the body’s center of gravity over the base of support while standing, walking, or performing other daily activities [1]. Muscle strength and power also play a critical role in the postural adjustment that occurs in response to external forces, which helps maintain balance [2]. The ankle joint is responsible for maintaining balance during standing, walking, and other activities. The ankle muscles work together to maintain balance and provide postural control [3,4,5,6]. The ankle strategy is the first line of defense in maintaining balance when the body is subjected to external forces [7,8]. In a prior investigation, Goncalves et al. discovered that individuals with moderate knee osteoarthritis experienced a reduction in calf muscle strength in comparison to a control group without knee osteoarthritis [9]. Similarly, Cattagni et al. conducted a comparative study on ankle muscle strength during early adulthood and in the elderly with and without a history of falling. The findings revealed that shifting in the position of the center of pressure (CoP) was more significant in older adults with a history of falls [10]. Recent research has revealed that elderly individuals with a history of falls exhibit a significantly lower maximum isometric contraction of their ankle muscles compared to those without a fall history [10]. These results imply that there is a decline in ankle muscle strength in the senior population, which can increase the risk of falling [11].

In current research, assessing muscle strength is considered essential in evaluating the risk of falls in elderly individuals. There are various methods available for this purpose, including the manual muscle test (MMT) [12], which is commonly used in clinical settings due to its ease of use and lack of equipment requirements [13]. However, MMT is less sensitive at reflecting changes in patients’ strength, and its results may be dependent on the experience and proficiency of the assessor, making inter-assessor comparisons unreliable [14]. On the other hand, hand-held dynamometers (HHDs) are a reliable and convenient tool for assessing muscle strength in the community-dwelling elderly population (r = 0.97) [15]. Although HHDs are portable and user-friendly, they can be costly. Meanwhile, isokinetic dynamometers provide the most accurate information, though their large size and high cost make them too cumbersome to carry around.

Nonetheless, there are also muscle strength tests that are convenient and do not require extensive equipment. Despite the existing evidence, there is a scarcity of references in the literature regarding field tests that specifically assess ankle muscle strength and power in the older population. Furthermore, the relationship between these measures and the decline in mobility and/or functionality remains understudied. In the assessment of ankle PF function in the elderly population, several field tests are commonly employed. These include the 10-m walk test [16], the timed up and go test [17], the stair climb test [18], and the 30-s chair stand test [19,20]. Despite their frequent utilization, further research is needed to ascertain the reliability, validity, and association of these field tests with mobility decline and functionality in the elderly. One such test is the standing heel-rise test (SHRT), a functional test that is suitable for evaluating the strength of the ankle PF muscles. Despite its level of difficulty for the function of the ankle PF muscles in the elderly, Andre et al. discovered that the SHRT is a useful indicator of ankle strength in older adults and can be used to assess the efficacy of an exercise program to prevent decreased mobility in the elderly [21]. This test requires less time to administer and can help minimize the risk of injuries or muscle fatigue, which are common concerns among the elderly and can have a positive impact on the subject. The SHRT demonstrates satisfactory levels of validity and reliability, making it a valuable measure of ankle strength in older adults. This test holds promise as a reliable indicator of ankle strength in this population [21,22,23]. Moreover, the single-leg balance test primarily assesses an individual’s ability to maintain postural stability; it also involves the recruitment and activation of various lower limb muscles, including the ankle plantar flexors. The ability to maintain balance on a single leg requires adequate strength and control of the ankle plantar flexor muscles [24,25]. However, the test conducted in the abovementioned study was unable to provide a quantitative prediction of the forefoot pedaling force strength. The availability of an equation to calculate the force based on this test would greatly benefit communities with significant populations requiring comprehensive testing. Hence, the research inquiry emerges as to whether it is feasible to identify an equation that can predict ankle PF muscle strength using variables derived from functional tests, balance assessments, and personal demographic data. The authors formulated the hypothesis that ankle PF muscle strength is likely to exhibit correlations with functional tests, balance assessments, and personal demographic data and that these variables collectively contribute to a multicorrelation that can be utilized to construct a predictive equation.

Therefore, the objective of this study was to investigate the correlation between ankle PF muscle strength and variables obtained from the SHRT. The SHRT was selected due to its simplicity and ease of administration, as it can be performed using easily accessible tools, such as stopwatches, which can lead to cost savings. Moreover, the SHRT can be effectively utilized in elderly individuals residing in community settings. Additionally, the aim of this research was to formulate an equation to estimate ankle muscle strength by utilizing the basic physical characteristics of the subject and variables related to their ability to perform the SHRT using stepwise multiple regression analysis. The novel findings of this study revolve around the development of a predictive equation for ankle muscle strength by examining the correlation between ankle flexor strength and SHR performance in healthy elderly individuals. This study’s outcomes provide a unique contribution to the existing academic literature by demonstrating that the SHRT is a feasible predictor of ankle muscle strength and balance in healthy elderly individuals. Moreover, the predictive equation established in this study can be utilized as a simple and practical tool for evaluating ankle strength and balance in older adults in both clinical and research settings. By establishing a connection between SHR performance, balance, and ankle muscle strength, this academic research holds the potential to facilitate the development of a new approach. In the event that this research yields hypothetical results, it would potentially enable healthcare professionals to assess ankle PF muscle strength in force units. Such an outcome would facilitate the implementation of timely and tailored rehabilitation programs for the elderly population. This, in turn, would allow for effective monitoring and the appropriate design of interventions for addressing ankle PF muscle strength in older adults.

## 2. Materials and Methods

### 2.1. Participants

This study is characterized as cross-sectional analytical research with a correlational study design in the context of developing a prediction equation. A total of 154 elderly participants were recruited through outreach efforts involving community leaders and community healthcare volunteers. Following the application of inclusion/exclusion criteria, 138 subjects met the specified criteria, while 16 subjects were excluded due to not meeting the inclusion/exclusion criteria. Among those excluded, 12 participants had knee osteoarthritis, 2 had rheumatoid arthritis, and 2 had a history of stroke. Consequently, out of the initial 138 subjects, 132 were available for analysis. Therefore, the study involved the participation of 132 elderly participants, aged 60 years or older, with a mean age of 67.30 ± 7.60 years, living in Muang District, Phayao Province, Thailand. To determine the appropriate sample size, we utilized the Correlation: Bivariate normal model G*Power analysis program, with a low correlation (r) value of 0.30, alpha = 0.05 and power = 0.95. The participants who met the inclusion criteria, which included elderly males and females who were healthy or individuals with chronic illnesses with controllable symptoms, such as diabetes and hypertension, and who were able to walk on their own without using a walking aid, were included in the study and are listed in Table 1. However, individuals with musculoskeletal problems of the lower extremities, such as osteoarthritis and rheumatoid arthritis, broken or dislodged bones, problems with the nervous system that affect balance and muscle strength, such as stroke and spinal cord disease, Parkinson’s disease, and those with problems related to communication, vision, and hearing, were excluded. The study received approval from the Human Research Ethics Committee at the University of Phayao (No. 2/168/60).

### 2.2. Research Protocol

The participants in the study were fully informed of the study’s purpose and data collection procedure by the researcher. Prior to participation, the participants were required to sign a consent form. Personal information was collected from the participants, including gender, age, body weight, height, and mid-calf circumference. Before the trial began, the participants were given five minutes to practice the movements. Once they became familiar with the movements, the experiment was officially carried out. The maximum voluntary contraction (MVC) of the ankle PF, SHRT, and single-leg standing balance test were measured with at least five minutes of rest provided between each test. The variable measurement sequence and method followed were as follows:

#### 2.2.1. The Maximum Voluntary Contractions (MVCs) Test of the Ankle PF

**Figure 1:**
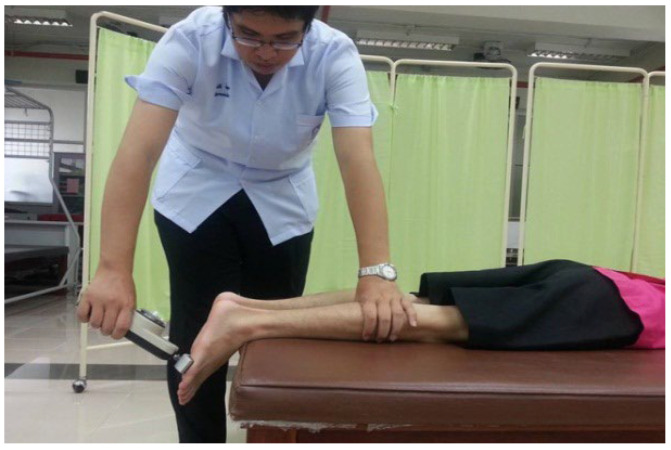
Push–pull dynamometer measurement of the maximum contraction force of the PF muscle.

Prior to the commencement of the test, the participants were advised by the assessor to stretch their calf muscles. The participants were then instructed to position themselves in a prone position on the bed with their feet extended over the edge. The push pads of the push–pull dynamometer (Baseline^®^ Analog Hydraulic Push-Pull Dynamometer, USA) were adjusted and placed on the ball of the foot to be measured, as illustrated in Figure 1. To familiarize the subjects with the test, they were allowed to perform 1 trial of submaximal contraction. The researcher then instructed the subjects to push their toes against the push pads of the push–pull dynamometer with maximal force and sustain the contraction for 4 s [26]. The test comprised three trials with a 2-min rest interval between each trial. The muscle contraction force variable was determined as the highest value obtained from the three trials. The MVCs tests are quantified in kilograms. This test for maximum contraction force of the ankle PF muscle was found to be highly reliable (ICC = 0.77–0.94) [27].

#### 2.2.2. Standing Heel-Rise Test (SHRT)

**Figure 2:**
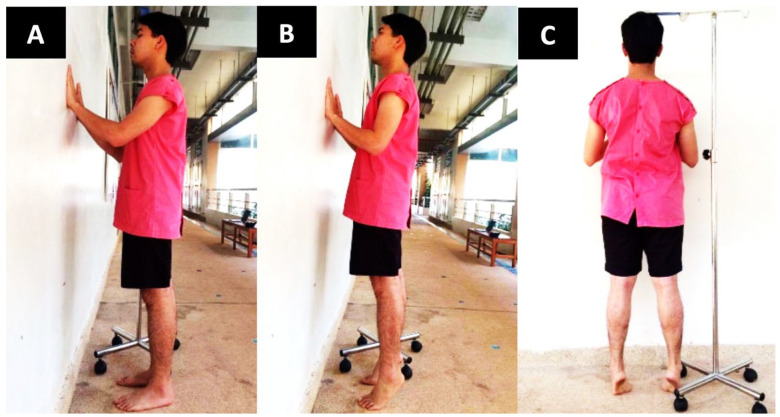
The SHRT, where (**A**) denotes the lateral view of starting position and (**B**,**C**) indicate lateral and back view of the maximum heel-rise position, respectively.

All the participants were instructed to stand in a specific position with both feet flat on the floor, shoulder-width apart, hands on the wall with slightly bent elbows, a straight back, and straight knees. Each participant was then asked to tiptoe on both feet simultaneously, with the goal of touching their head to a wooden board placed at their maximum tiptoe height. They were required to touch the wooden board and then place their heels back on the floor before continuing 5 times as quickly as possible, as shown in Figure 2 [21]. The test was conducted over 3 trials, with a 3-min rest period between each cycle. The variable of this test was the time taken to complete the test. In addition, the power index of the SHRT was calculated using the following formula: power = work/time [28], as follows:

$$\mathrm{Power}\;\mathrm{index}\;\mathrm{of}\;\mathrm{SHRT}\;(\mathrm{Nm}/s)=\begin{array}{c} \mathrm{Body}\;\mathrm{weight}\times 9.81\times 0.05\times 5\\ \mathrm{Time}\;\mathrm{taken}\;\mathrm{to}\;\mathrm{complete}\;\mathrm{the}\;\mathrm{SHRT} \end{array}$$

Please note that the value of 0.05 represents the distance in meters from the starting point to the end of the tiptoe, and the value of 5 refers to the number of occasions the SHRT is performed. The calculation formula for the power index in this study is derived from the fundamental physics power formula of work divided by time. The work component is based on the vertical displacement of the body’s center of mass, which is set at 0.05 m per repetition. This value is multiplied by the number of repetitions (5 times) and the acceleration due to gravity (9.81 m/s^2^). The resulting value is then divided by the time taken to complete the short heel-rise test (SHRT). This calculation approach draws inspiration from the power index calculation used in the five-time sit-to-stand test, as described in the study conducted by Takai et al. [29]. The reliability of the standing heel-rise test was assessed using the intraclass correlation coefficients (ICC), yielding values ranging from 0.85 to 0.99 [22,23]. These high ICC values indicate a strong level of reliability for the test. Additionally, the correlations between the three muscle strength tests demonstrated evidence of convergent validity, with correlation coefficients ranging from 0.56 to 0.66 [23]. These findings suggest that the tests are measuring similar constructs of muscle strength, further supporting their validity.

#### 2.2.3. Single-Leg Standing Balance Test

The experimental procedure for the single-leg standing balance test commenced with the participants assuming a standing position with both legs on the ground and eyes gazing straight ahead. The researcher then instructed the subjects to shift their weight onto their dominant leg while crossing their arms in front of their chest. The non-dominant knee was flexed at a 90-degree angle, and a timer was initiated to measure the duration of time the participants maintained balance for as long as possible [30]. The test was conducted three times, with each test separated by a 3-min interval. Two single-leg standing balance testing conditions were tested in this study, including eyes open (SSEO) and eyes closed (SSEC), respectively.

### 2.3. Statistical Analysis

The data distribution was assessed using the Shapiro–Wilk test, which revealed that the variables demonstrated a normal distribution. Descriptive statistics were employed to provide a characterization of the subjects, while the Pearson product-moment correlation coefficient was employed to determine the correlation coefficient between the maximum ankle plantar flexion contraction force, the time taken to complete the test, and the power index of the SHRT. The stepwise multiple linear regression analysis technique was applied to conduct a multiple regression analysis and develop an ankle PF strength prediction equation. This equation was based on several variables, including the subject’s demographic information, the time taken to complete the test, and the power index of the SHRT. The best model based on the highest adjusted R^2^ value and the lowest degree of variance inflation was selected. To identify the most significant independent variable coefficients for each prediction model, we examined their significance within the model. All the statistical analyses were conducted using SPSS version 21 (SPSS Inc., Chicago, IL, USA), and a significance level of 0.05 was employed for all the statistical tests.

## 3. Results

A sample of 132 elderly individuals were enrolled in this study, consisting of 57 males and 75 females with a mean age of 67.30 ± 7.60 years. The participants’ mean mass was 58.60 ± 10.94 kg, their mean height was 157.99 ± 12.87 cm, and their mean body mass index (BMI) was 23.38 ± 3.90 kg/m^2^. The calf circumference of the participants was 32.13 ± 4.10 cm. The maximum ankle PF muscle strength was found to be 26.47 ± 9.35 kg, while the average time taken to complete the SHRT in five repetitions was 4.75 ± 1.41 s. Additionally, the power index of the SHRT, calculated from the body weight and time taken to complete the test, had an average value of 32.34 ± 9.81 Nm/s. The participants’ average single-leg standing balance test duration with eyes open (SSEO) was 29.30 ± 30.02 s, while the duration with eyes closed (SSEC) was 5.29 ± 4.84 s, as shown in Table 1.

**Figure 3:**
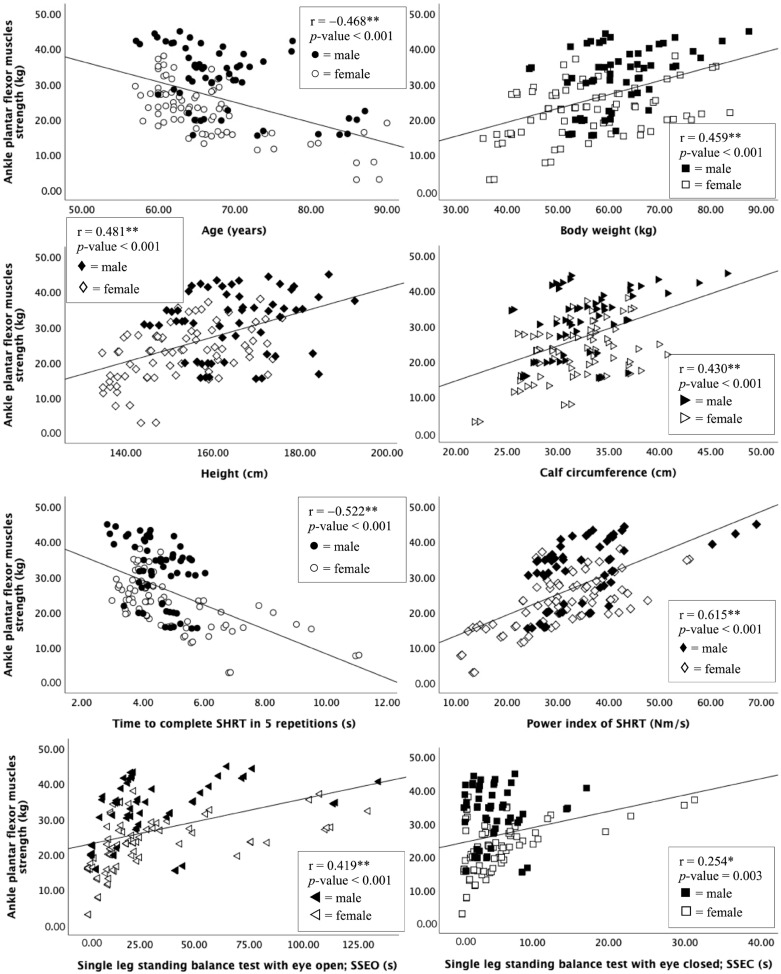
Scatter plots showing relationship between ankle PF strength variable and demographic information of the participant, single-leg standing balance, and SHRT performance. Note: ** Correlation is significant at *p* < 0.001, * Correlation is significant at *p* < 0.005.

The strength of ankle PF muscles was examined in relation to the participants’ demographic characteristics, including gender, age, weight, height, and calf circumference, revealing moderate levels of correlation (r = 0.430 to 0.481, *p* < 0.001). A moderate negative correlation (r = −0.522, *p* < 0.001) and a moderate positive correlation (r = 0.615, *p* < 0.001) were also discovered between the ankle PF muscle strength and time to complete the SHRT throughout 5 repetitions and the power index of the SHRT, respectively. Additionally, ankle PF muscle strength was found to be weakly correlated with the SSEO (r = 0.419, *p* < 0.001) and SSEC (r = 0.254, *p* < 0.005). Table 2 and Figure 3 present the correlation findings.

Based on the results of a multiple regression analysis of the ankle PF strength, all five models of the factors were identified and are presented in Table 3. Model 1 showed that only the power index of the SHRT factor had a significant effect on ankle PF strength. Meanwhile, models 2, 3, 4, and 5 included gender, age, SSEO, and calf circumference, respectively. Among all five models, model 5 showed a significantly strong correlation (r = 0.816, *p* < 0.05) and had the highest coefficient of determination (R^2^ = 0.653), indicating that the combined effect of the power index of the SHRT, gender, age, SSEO, and calf circumference accounted for 65.3% of the variance in the ankle PF strength. The standard error of estimation was approximately 5.51 kg.

As a result, the equation for the ankle PF strength’s predictive accuracy was 24.31 − 0.20(A) + 8.14(G) + 0.49(CC) + 0.07(SSEO) + 0.20(BW/t-SHRT) ± 5.51 (kg), where A represents age, G stands for gender (male = 1, female = 0), CC represents the calf circumference, SSEO represents the single-leg standing balance test with eyes open, BW represents body weight, and t-SHRT represents the time taken to complete the SHRT.

## 4. Discussion

As individuals age, they undergo a natural and intricate process that impacts their physical and physiological functioning in diverse ways. Notably, a prominent manifestation of aging is the decline in muscle mass and strength, referred to as sarcopenia [31]. The prevalence of sarcopenia is a major public health issue, as it is linked to elevated rates of disability, falls, and mortality among older adults [32]. Research findings reveal that as individuals age, their muscle strength undergoes a progressive decline that commences in the third decade of life and persists throughout their lifespan. Muscle strength deteriorates by approximately 15% per decade after the age of 50, and the decline is even more pronounced after 70 years. Multiple factors contribute to this decrease, including a reduction in muscle mass, modifications in muscle fiber types, and changes in the neuromuscular system [33].

The decline in muscle strength associated with aging poses a significant risk for falls in older adults, with age-related muscle loss or sarcopenia, leading to reduced muscle mass, strength, and power, ultimately resulting in decreased balance and stability [34]. Consequently, the likelihood of falls increases, with potentially serious consequences, such as fractures, head trauma, and hospitalization. Several studies have established the correlation between reduced muscle strength and the risk of falls in older adults [35].

As people age, ankle muscle strength and balance become crucial for maintaining mobility and functional independence [18,36,37]. A decline in ankle muscle strength and proprioception can cause instability [38] and balance impairment [24,25] and can increase the risk of falls in older adults [11]. Various studies have emphasized the significance of ankle muscle strength and balance in older adults. Chung et al. (2022) reported that ankle dorsiflexion strength was a significant predictor of balance and gait speed in older adults [39]. Spink et al. (2010) revealed that ankle plantar flexion strength was associated with balance and functional capacity in older women [27]. Furthermore, interventions that target ankle muscle strength and balance have been shown to improve mobility and functional outcomes in older adults [40,41,42]. Therefore, maintaining ankle muscle strength and balance is crucial for older adults to remain mobile and independent and prevent falls.

Ankle muscle strength can be assessed in various ways among older adults, each with its own advantages and limitations. The three most commonly used methods are isokinetic dynamometry, hand-held dynamometry, and the one-repetition maximum (1RM) test [43]. Isokinetic dynamometry is considered the gold standard for measuring ankle muscle strength due to the fact that it provides precise and objective measurements of the force generated by the ankle muscles. However, it is costly and requires trained personnel to administer the test [44]. It is important to consider the advantages and limitations of each method when assessing ankle muscle strength in older adults.

The standing heel-rise test (SHRT) is a simple, non-invasive functional test employed to evaluate the strength of the ankle PF muscles in older adults. The test involves standing with both feet flat on the ground and then rising up onto the balls of the feet as high as possible [21]. The number of successful repetitions performed within a certain time frame is used as an indicator of ankle PF muscle strength [21]. The SHRT has been found to be a reliable and valid measure of ankle PF muscle strength in older adults and has been applied in clinical and research settings to evaluate muscle strength, balance, and mobility [22,45]. The test has also been used as an outcome measure in studies investigating the effects of various interventions, such as exercise and balance training on lower extremity function in older adults.

The purpose of this study was to determine the strength of the ankle PF muscles and to find an equation to calculate the strength of these muscles. The relationship between the strength of the push–pull dynamometer and the ability to perform the SHRT was investigated. Additionally, other factors that may affect the strength of the ankle PF muscles were examined, such as demographic variables like gender, age, weight, and calf circumference. The study revealed that a prediction equation could be developed to calculate the strength of the PF muscle based on the force measured during the SHRT and the demographic variables. It is a well-established relationship between ankle PF muscle strength and age, gender, weight, balance, and calf circumference. Based on the regression analysis and the interpretation of coefficients, the influence of various variables on ankle PF muscle strength was observed. Specifically, it was found that sex had a significant impact on strength. Males exhibited approximately 8 kg greater ankle PF muscle strength compared to females. Additionally, for every 1 unit increase in the power index by the SHRT (kg/m^2^), the stability by SSEO (s), and the calf circumference (cm), there was an associated increase in ankle PF muscle strength by 0.204 kg, 0.067 kg, and 0.490 kg, respectively. Conversely, for every 1-year increase in age, there was a decrease in ankle PF muscle strength by 0.381 kg. These findings highlight the direction and magnitude of the influence that each variable exerts on ankle PF muscle strength. As individuals age, their ankle PF muscle strength tends to decline, which can lead to reduced mobility and increased risk of falls [46]. Gender differences have also been observed, with men generally exhibiting greater strength than women [47]. Body weight has been shown to influence ankle PF muscle strength, with greater body weight typically resulting in greater strength [48]. Nevertheless, excessive weight can also lead to reduced strength due to increased strain on the muscles and joints [49]. Balance is another important factor, as it affects the ability to maintain posture and stability during movement. Individuals with poorer balance tend to exhibit weaker ankle PF muscles, which can further exacerbate their balance problems [50]. Calf circumference is also linked to ankle PF muscle strength, as a larger circumference indicates a greater muscle mass and, therefore, greater strength [51]. However, calf circumference alone may not be a reliable predictor of strength, as other factors such as age, gender, and weight also play a role.

This equation provides a unit of measurement for the actual force produced by the ankle PF muscle, similar to the measurement provided by force-measuring equipment. The equation of the ankle PF muscle strength is 24.31 − 0.20(A) + 8.14(G) + 0.49(CC) + 0.07(SSEO) + 0.20(BW/t-SHRT) ± 5.51 (kg), where A represents age, G stands for gender (male = 1, female = 0), CC represents the calf circumference, SSEO represents the single-leg standing balance test with eyes open, BW represents body weight, and t-SHRT represents the time taken to complete the SHRT. The prediction equation was found to have moderate predictive ability, yielding a prediction accuracy of roughly 65.3%, with a corresponding measurement error of approximately 5.51 kg. The utilization of a prediction equation in this research study resulted in the assessment of ankle PF muscle strength in a significant number of elderly individuals in the community. The benefit of using the prediction equation lies in its ability to provide an accurate and efficient means of measuring muscle strength, which can aid in the diagnosis and treatment of age-related muscle weakness and functional decline. One potential clinical implication is the utilization of estimated ankle PF strength as an objective measure to assess functional capabilities in individuals. By incorporating this estimation into clinical assessments, healthcare professionals can gain valuable insights into a patient’s lower limb strength and overall physical performance. This information can guide treatment planning, goal setting, and the monitoring of progress over time. Moreover, the findings of this study can inform the development or modification of interventions targeting ankle muscle strength. With the estimated ankle PF strength as a baseline measure, healthcare providers can tailor rehabilitation or exercise programs to address specific weaknesses or imbalances in ankle strength. These interventions may involve targeted exercises, resistance training, or other therapeutic modalities aimed at improving ankle muscle strength and functional outcomes. Although a predictive equation for estimating ankle PF strength is presented, validation would enable an assessment of the equation’s predictive accuracy in future studies. By comparing the predicted ankle PF strength values obtained from the equation with the actual measured values in independent samples, researchers can determine how well the equation performs. This analysis would shed light on the equation’s reliability and precision, informing potential refinements or adjustments to improve its accuracy.

Although the present study provides valuable insights, it is essential to acknowledge the limitations of the study. One of the limitations is the measurement of ankle PF muscle strength using a push–pull dynamometer, which is not considered the gold standard method, unlike the isokinetic dynamometer. The push–pull dynamometer measures the maximum form of force produced by the muscle’s isometric contraction. Nevertheless, it is still widely used for measuring muscle force due to its accessibility and accuracy. As a result, future studies should investigate the relationship between the SHRT performance and machine-measured ankle muscle strength using the gold standard isokinetic dynamometer to address this limitation. Moreover, in this study, a sample of participants exclusively from a specific geographical area was utilized, which raises concerns about the generalizability of the findings to other populations or regions. It is important to acknowledge this limitation when interpreting and applying the results to broader contexts. One of the limitations of this study is the inclusion of participants with chronic illnesses, such as diabetes and hypertension, which have controllable symptoms. However, relying solely on the self-reporting of these conditions introduces a potential risk of misclassification or underreporting. It is important to consider this limitation when interpreting and drawing conclusions from the findings. An analysis of additional factors that can potentially influence ankle PF muscle strength, including the participants’ physical activity level, nutritional status, and medication use, among others, is highly significant and warrants attention. Consequently, it is recommended that future studies address these factors to further enhance our understanding in this area of research. Exploring these aspects would contribute valuable insights into the field and deepen our comprehension of the various determinants of ankle PF muscle strength. Other limitations include that data on the fall history or fall risk of elderly participants were not gathered and certain demographic groups were not excluded, such as individuals with certain medical conditions or disabilities, which prevented the investigation of the relationship between PF muscle strength and fall risk and specific demographic data. The findings may not fully capture the diversity of the target population, limiting the generalizability and external validity of the study. In future research, incorporating data collection on fall history and risk and certain medical conditions or disabilities is imperative. Nevertheless, the authors of this study were able to obtain data on balance ability, which enabled some correlation with falls.

## 5. Conclusions

In conclusion, in the present study, several factors that have a significant impact on the strength of the ankle PF muscles were identified, including age, gender, weight, calf circumference, and SHRT performance. Based on our findings, we were able to develop a predictive equation to estimate ankle PF strength, which provides a useful tool for clinicians and researchers to estimate the strength of ankle PFs as well as assess the effectiveness of interventions aimed at improving this important aspect of lower limb function.

## Figures and Tables

**Table 1:** Subject demographic data and anthropometric characteristics variables.

Variables (*n* = 121)	Mean ± SD
Gender (males/females)	57/75
Age (years)	67.30 ± 7.60
Mass (kg)	58.60 ± 10.94
Height (cm)	157.99 ± 12.87
Body mass index; BMI (kg/m^2^)	23.38 ± 3.90
Calf circumference (Centimeter)	32.13 ± 4.10
Ankle PF muscles strength (kg)	26.47 ± 9.35
Time to complete SHRT in 5 repetitions (s)	4.75 ± 1.41
Power index of SHRT (Nm/s)	32.34 ± 9.81
Single-leg standing balance test with eye open; SSEO (s)	29.30 ± 30.02
Single-leg standing balance test with eye closed; SSEC (s)	5.29 ± 4.84

**Table 2:** Correlation between ankle PF strength variable and demographic information of the participant, single-leg standing balance, and SHRT performance.

		Ankle PF Muscle Strength
**Demographic data Variables**	Gender	0.479 ** (*p*-value < 0.001)
	Age	−0.468 ** (*p*-value < 0.001)
	Weight	0.459 ** (*p*-value < 0.001)
	Height	0.481 ** (*p*-value < 0.001)
	BMI	0.199 * (*p*-value = 0.022)
	Calf circumference	0.430 ** (*p*-value < 0.001)
**Time to complete SHRT in 5 repetitions**		−0.522 ** (*p*-value < 0.001)
**Power index of SHRT**		0.615 ** (*p*-value < 0.001)
**Single-leg standing balance test with eye open; SSEO**		0.419 ** (*p*-value < 0.001)
**Single-leg standing balance test with eye closed; SSEC**		0.254 * (*p*-value = 0.003)

Note: ** Correlation is significant at *p* < 0.001, * Correlation is significant at *p* < 0.005.

**Table 3:** Model of regression analysis for ankle PF muscle strength with different predictive variables.

Model	Included Variables	*β*	*p*-Value	r	Adjusted r^2^	SEE
1	Constant	7.524	0.001 *	0.615	0.374	7.399
	Power index of SHRT	0.586	<0.001 **			
2	Constant	7.069	0.001 *	0.708	0.494	6.650
	Power index of SHRT	0.510	<0.001 **			
	Gender	6.774	<0.001 **			
3	Constant	42.427	<0.001 **	0.783	0.604	5.881
	Power index of SHRT	0.352	<0.001 **			
	Gender	8.290	<0.001 **			
	Age	−0.459	<0.001 **			
4	Constant	37.283	<0.001 **	0.797	0.624	5.734
	Power index of SHRT	0.325	<0.001 **			
	Gender	8.142	<0.001 **			
	Age	−0.391	<0.001 **			
	SSEO	0.051	0.006 *			
5	Constant	24.310	0.001 *	0.816	0.653	5.507
	Power index of SHRT	0.204	0.003 *			
	Gender	8.138	<0.001 **			
	Age	−0.381	<0.001 **			
	SSEO	0.067	<0.001 **			
	Calf circumference	0.490	0.001 *			

Note: ** Correlation is significant at *p* < 0.001, * Correlation is significant at *p* < 0.005, SEE is standard error of estimation, Gender was coded as a binary variable, with female = 0 and male = 1.

## Data Availability

The data are unavailable due to ethical restrictions.
